# Definition of the Metagenomic Profile of Ocean Water Samples From the Gulf of Mexico Based on Comparison With Reference Samples From Sites Worldwide

**DOI:** 10.3389/fmicb.2021.781497

**Published:** 2022-01-28

**Authors:** Antonio Loza, Fernando García-Guevara, Lorenzo Segovia, Alejandra Escobar-Zepeda, Maria del Carmen Sanchez-Olmos, Enrique Merino, Alejandro Sanchez-Flores, Liliana Pardo-Lopez, Katy Juarez, Rosa-Maria Gutierrez-Rios

**Affiliations:** Instituto de Biotecnología, Universidad Nacional Autónoma de México, Cuernavaca, Mexico

**Keywords:** Gulf of Mexico, reference metagenomes, metabolic potential, metabolic network, key enzymes, hydrocarbon degradation

## Abstract

Computational and statistical analysis of shotgun metagenomes can predict gene abundance and is helpful for elucidating the functional and taxonomic compositions of environmental samples. Gene products are compared against physicochemical conditions or perturbations to shed light on the functions performed by the microbial community of an environmental sample; however, this information is not always available. The present study proposes a method for inferring the metabolic potential of metagenome samples by constructing a reference based on determining the probability distribution of the counts of each enzyme annotated. To test the methodology, we used marine water samples distributed worldwide as references. Then, the references were utilized to compare the annotated enzymes of two different water samples extracted from the Gulf of Mexico (GoM) to distinguish those enzymes with atypical behavior. The enzymes whose annotation counts presented frequencies significantly different from those of the reference were used to perform metabolic reconstruction, which naturally identified pathways. We found that several of the enzymes were involved in the biodegradation of petroleum, which is consistent with the impact of human hydrocarbon extraction activity and its ubiquitous presence in the GoM. The examination of other reconstructed pathways revealed significant enzymes indicating the presence of microbial communities characterizing each ocean depth and ocean cycle, providing a fingerprint of each sampled site.

## Introduction

The Gulf of Mexico (GoM) is an area with important oil industry activity that is constantly affected by the extraction and transportation of hydrocarbons, sometimes causing large-scale spills, which have repeatedly disturbed the area (Jernelv, 2010; Michel et al., 2013; Soto et al., 2014). The introduction of petroleum in fragile marine environments can result in severe ecological perturbations. Crude oil is composed of thousands of components, which can be mainly separated into saturates, aromatics, resins, and asphaltenes. The aromatic compounds are subject to physicochemical modifications, providing the environment with smaller-molecular-weight products that are readily biodegraded in marine environments. Other compounds such as resins and asphaltenes are resistant to biodegradation (Harayama et al., 1999). In the environment, petroleum biodegradation is carried out by microorganisms (Head et al., 2006), and the isolation of those microorganisms capable of degrading petroleum components is therefore an area of interest. However, given the impossibility of identifying and isolating many of these organisms, methodologies based on the sequencing of DNA recovered from environmental samples are now a common approach.

Shotgun metagenomics has become an essential tool for inspecting the metabolic potential and taxonomic composition of environmental DNA (Gilbert and Dupont, 2011; Sharpton, 2014; Bharti and Grimm, 2021). Sequencing technology for performing such analyses has significantly evolved in recent decades, making it possible to inspect the composition of samples representing a vast collection from distinct environments (Gilbert and Dupont, 2011; Land et al., 2015; Wang et al., 2017). Nonetheless, even with the improvements in sequencing technologies, it is difficult to obtain comparable and reproducible results with many of them. This is linked to other challenges, including study design, data access, metadata standardization, and the use of analysis tools that should be improved to enable comparative metagenomics in different projects worldwide.

Several studies outlining the functional potential of ocean water columns and sediments are based on metagenomic studies. The metabolic potential of the microbial communities revealed in metagenomic studies is expected to correlate with the characteristics of the physical and chemical parameters of each sample. Examples of these measurable parameters are dissolved organic matter (DOM), inorganic matter, temperature, pH, and depth. Other parameters that are difficult to assess are the direct and indirect interactions between microorganisms distributed in water layers and sediments. The inference of pathways based only on the above information may make some functions challenging to appraise since these parameters are not always available.

In recent years, publicly available metagenomic databases have grown to house thousands of studies, providing the unique opportunity to compare experiments to identify the differences between gene products and taxa in distinct biomes (Paczian et al., 2019; Mitchell et al., 2020). However, these comparisons represent a great challenge due to the variation in experimental designs, the use of different sequencing platforms, and the posttreatments applied to sequences. Therefore, it is necessary to develop new methodologies for comparing this information. The observations mentioned above prompted us to determine whether it was possible to develop a method by which to infer the metabolic potential of shotgun metagenomic samples in a comparative manner to help address the lack of specific information concerning environmental conditions from which the samples were extracted. For this purpose, the proposed method requires a selection of metagenomes serving as a reference to identify the probability distribution describing the behavior of each enzyme into the reference. The distribution of each enzyme will enable the determination of whether the same enzyme in different metagenomes presents an atypical behavior with respect to the reference. For this work, the reference enzymes were obtained from annotated metagenomes of water samples from oceanographic campaigns distributed worldwide for comparison with water samples from the GoM. The enzymes identified as atypical (overrepresented) were used to reconstruct metabolic pathways. We evaluated the consistency of the results considering the origin in the GoM and the depth in the water column. We also discuss the implications of the type of probability density function (PDF) found for the enzymes, paying special attention to those defined as essential to life. We observed lower dispersion of essential enzymes relative to enzymes expected to be less preserved in all organisms. Moreover, we found that the distribution of a group of aminoacyl tRNA synthetases cataloged as essential presented long tails, a result that appeared to be consistent with other findings based on the unequal distribution of some of them even within the same taxon. Finally, we were able to identify relevant pathways related to hydrocarbon degradation and their connection with other metabolic pathways incorporating the end products. We also found relevant enzymes and pathways related to ocean cycles and the niche from which the samples were taken. We concluded that even with the limitations of metagenomic techniques, this approach has great potential for inferring broader functional profiles of any biome by taking advantage of the number of samples currently available in public repositories.

## Materials and Methods

### Data Collection and Processing

#### Gulf of Mexico Samples

**FIGURE 1:**
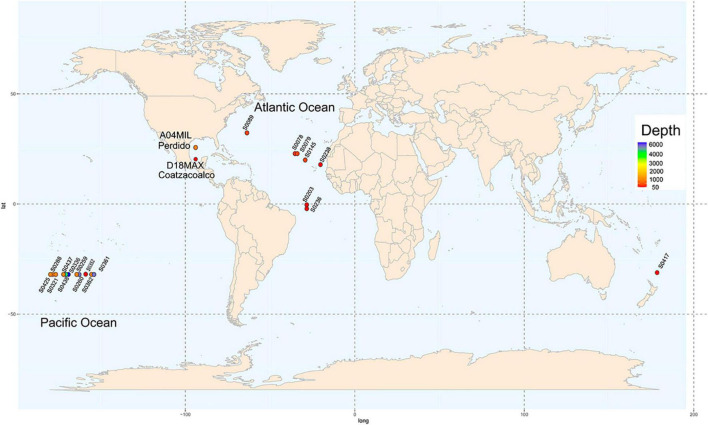
Sampling locations. Nineteen locations water sample collection in the GEOTRACES project distributed worldwide are shown in circles. Depth from shallow to deep is represented in colors ranging from red to purple the map shows two sampling locations in the Gulf of Mexico, in the Perdido Fold Belt and Campeche Knolls.

We evaluated two metagenomes of water samples from the GoM collected in March 2016 (Figure 1; Raggi et al., 2020). Sample A04 was found at a depth of 1,000 m in the Antarctic Intermediate Water (AAIW) of the Perdido Fold Belt (NW; coordinates, 25°52′47.0″N 94°40′07.0″W). Sample D18 was collected at Campeche Knolls, Coatzacoalcos (SE), in the maximum fluoresce layer at a depth of 76 m at 19°55′52.1″N 94°20′26.4″W. The samples were sequenced and processed as described previously (Raggi et al., 2020), except for the annotation step performed using the MG-RAST suite (Paczian et al., 2019). In Supplementary Table 1, we show the MG-RAST identifiers assigned to each metagenome.

#### Reference Samples

We downloaded the assembled sequences of 19 water samples from the Sequence Read Archive (SRA) database collected on GEOTRACES cruises (Biller et al., 2018) covering sites in the Atlantic and Pacific Oceans and considering different depths (Figure 1). The assembly published by the GEOTRACES project was uploaded to the MG-RAST suite. Supplementary Table 1 shows the geographical coordinates, depths, sequence technology used, and MG-RAST and SRA identifiers of each sample.

The GoM and reference samples and the annotated gene products of enzymes showing 45% sequence identity and an *e*-value ≤ 1E−04 were classified based on their functions according to the Enzymatic Commission (EC) number (Tipton and Boyce, 2000). A minor group of enzymes with incomplete enzymatic activity descriptors, such as 1.-.-. -, 1.1.-. -, or 1.1.1.- were discarded when absent in the metabolic Kyoto Encyclopedia of Genomes and Genomes (KEGG) databases used in this study (Kanehisa et al., 2017).

### Metagenome Assembly, Binning, and Functional Annotation

The raw reads from D18_MAX and A04_AAIW with SRR11308320 and SRR11308318 SRA ID numbers, respectively, were first filtered using fastp^1^ with default parameters to trim adaptors and clean reads. Trimmed paired-end reads were then merged into a single fasta file for later assembly with IDBA-UD (Peng et al., 2011) with default settings. A scaffolding step was carried out on the contigs of the assemblies with OPERA (Gao et al., 2011). Bowtie v.2.3.5.1 (Langmead and Salzberg, 2012) was used to map sequence reads back to the assembly. Then, MetaBAT v.2 (Kang et al., 2019) was used to bin the assemblies under default settings. Next, to calculate the completeness, contamination, and strain heterogeneity of the bins formed, CheckM (Parks et al., 2015) was used. The taxonomic classification of the bins of every sample was performed with Phylophlan v.3.0 (Asnicar et al., 2020). Finally, the functional annotations of the bins were completed with evolutionary genealogy of genes: non-supervised Orthologous Groups (eggNOG mapper) (Huerta-Cepas et al., 2017).

### Statistical Analysis Inferring the Metabolic Potential

First, let us focus on a given element, *m*, of the reference metagenomes and on the subset of annotations that produce an enzyme as the output. Let *y*_*gm*_ denote the observed count of annotations for enzyme *g* in *m*. Now, we will assume that occurrences of each enzyme follow a multinomial or Poisson distribution. This assumption is justified because a few thousand different enzymes are typically annotated with relatively low frequencies. Let us imagine that we repeat the annotation process under the same conditions. In this case, we expect that, on average, the count of occurrences for a particular enzyme, *g*, per million annotated sequences can be calculated as follows:


λgm=ygmNm 106,

where *N_m_* is the total number of enzymes annotated in the entire metagenome *m*. However, some reflection results in the realization that, due to random effects throughout the annotation process, deviations from this value much larger than the expected variance can occur. To proceed to the metagenome analysis, we conclude that the distribution parameter λ_*gm*_ is not a constant but is sampled from a distribution.

We extend this idea further and consider a collection of metagenomes processed according to the same workflow but under slightly different environmental conditions. In this case, we expect greater variability in the observed *y*_*gm*_. Nevertheless, we can assume that this extra variability can be captured by theoretical distributions of the λ_*gm*_ parameters, which we have denoted with *f*(*x*; θ_*gl*_), where θ_*gl*_ represents certain parameters to be determined. Thus, for a given metagenome, *k*, not belonging to the reference, we can tell if the observed frequencies, *y*_*gk*_, are within the reference values or constitute a statistically unexpected event.

Once the parameters of each enzyme were assigned to each metagenome in the reference, we obtained an empirical distribution for the enzymes in the reference (Supplementary Table 2). We fitted these empirical distributions using the *fitdistr* function from the R package MASS^2^. Since the data to be fitted were frequencies, we try to fit a normal distribution only when $\underline{x}>3s$, where $\underline{x}$ and *s* are the sample means and standard deviation. When this condition was not fulfilled, the theoretical distributions with which we attempted to fit the data were gamma, log-normal, Weibull. In this case, the goodness of fit for each distribution was verified through the Anderson–Darling test in the R package *goftest*.^3^ To choose the optimal fit, we used the Akaike information criterion.

Next, for the two metagenomes under study, we calculated quantities in a manner analogous to Eq. 1:


(1) λgk=ygkNk 106,

with *k* = 1, 2, and using the set *f*_*g*_(*x*; θ_*gl*_) of fitted distributions, we calculated the probability:


(2) $$P_{gk}=P\left(X\leq \lambda_{gk}\right)=\int_{-\infty }^{\lambda_{gk}}dxf_{g}\left(x,\theta_{gl}\right)$$

**FIGURE 2:**
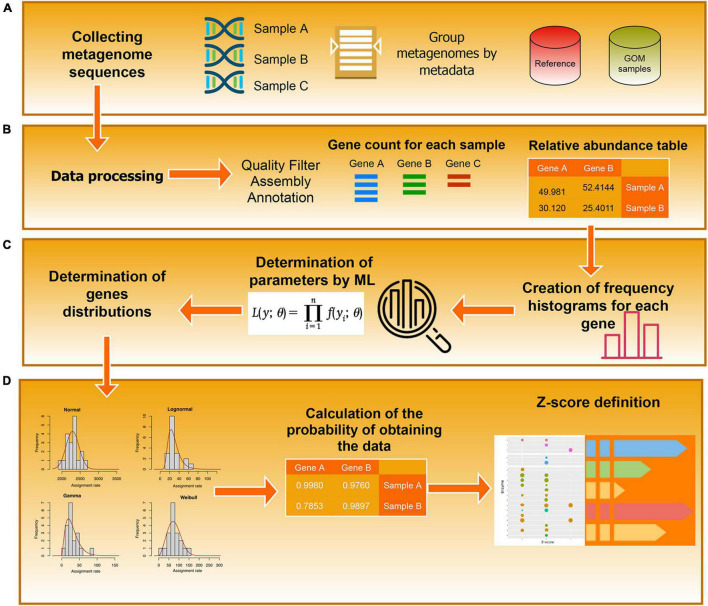
Workflow diagram. The diagram represents the steps used to determine the EZSs of the annotated enzyme. **(A)** Data collection. **(B)** Pre-processing. **(C)** Definition of the probability distributions **(D)** Calculation of the z-Scores.

To ease interpretation, rather than to work directly with the values in Eq. 2, we choose to express these probabilities in terms of an equivalent *Z*_*gk*_ score determined with the following equation:


(3) $$P_{gk}=\int_{-\infty }^{Z_{gk}}dxN\left(x\right),$$

where *N(x)* is the PDF of a normal distribution with a zero mean and a unitary standard deviation. The set {*Z*_*gk*_} is the basic information that we use to derive our results. An example of the results of these procedures is provided in Table 1, and the associated workflow is in Figure 2.

**TABLE 1:** Reference metagenomes and results of the GoM samples.

EC	1.1.1.35	1.14.13.7	1.3.1.32	2.8.3.8
M1	2104.258	21.69339	21.693386	65.08016
M2	2111.734	34.36254	28.114808	93.71603
M3	2213.504	28.22319	32.255073	76.6058
M4	2089.64	22.5907	22.590702	71.53722
M5	2594.575	33.75057	29.531753	42.18822
M6	2365.485	15.87574	12.70059	41.27692
M7	1939.527	38.25496	49.73145	38.25496
M8	2174.35	20.60995	41.219903	103.04976
M9	2206.178	15.68373	47.051197	151.60941
M10	2656.021	16.39519	2.732532	19.12772
M11	2541.855	52.83302	23.481344	70.44403
M12	2363.248	55.19262	85.297689	120.42027
M13	2349.213	25.28301	18.962259	63.20753
M14	2267.837	20.76774	33.228387	87.22452
M15	2315.387	64.46553	53.721273	118.1868
M16	2339.845	21.02557	15.018262	45.05479
M17	2412.827	20.0512	20.051197	66.83732
M18	2169.387	25.7647	15.458818	77.29409
M19	2317.505	28.00611	28.006105	98.02137
Distribution	Normal	Lognormal	Gamma	Weibull
p1	2291.17784	3.295168	2.683552	2.555658
p2	176.84802	0.4076202	0.08778131	86.05314
EZS (D18_MAX)	−0.220768	1.5988	1.14147	0.838235
EZS (A04_AAIW)	3.27996	2.18523	3.7638	2.8628

*The table illustrates the distributions and parameters (p1, p2) obtained from the rates of four enzymes (EC:) using the reference metagenomes (M). The EZSs calculated for each enzyme in the EZS (D18_MAX) and EZS (A04_AAIW) are also shown.*

### Global Metabolic Network

To reconstruct the global metabolic network, we retrieved the EC kgml files from all the metabolic pathways available in KEGG (Kanehisa et al., 2017). We did not include all kgml files from glycan biosynthesis and metabolism. This step was also useful for curating EC numbers that were not updated by the MG-RAST databases. With the use of a custom Python script, all the kgml pathways were merged into one global metabolic network. Then, for each metagenomic sample (A04_AAIW and D18_MAX), we built a subnetwork filtering out all EC numbers from the global metabolic network with *z*-scores lower than −1. The networks were displayed using Cytoscape software (Shannon et al., 1971).

### Global Network Clusters

For each sample’s metabolic network, we built clusters (groups of nodes more connected to each other than to the rest of the network) using MCL software (Van Dongen, 2008) with an inflation value of 1.5. Next, we filtered out all those clusters with fewer than four EC numbers. Finally, we counted the total number of intersecting ECs between each remaining cluster and all the KEGG pathways employed for global network construction.

## Results

### Generation of the Reference Enzymes and Equivalent *z*-Score Interpretation

The selection of genes encoding enzymes representing the metabolic potential of metagenomic samples is mainly based on the search of key (marker) enzymes of expected metabolic pathways of the microbial communities. The resolution of the reconstructed pathways is usually improved by searching for other enzymes using software and data bases as for example KEGG Mapper (Kanehisa et al., 2021) or by manual inspection.

In contrast, our approach aims to highlight enzymes, in a sample under study, with values of the rates in Eq. 1 that are significantly different from those of the reference group of metagenomes under similar environmental conditions. We then take this set of enzymes with atypical rates as a seed with which to reconstruct a metabolic network, which gives a hint of the metabolic potential of the sample.

**FIGURE 3:**
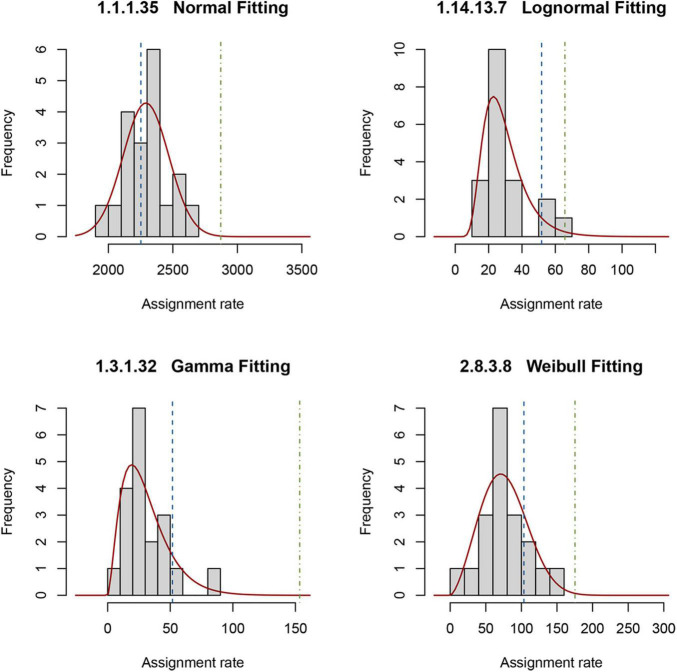
Probability density functions of the reference metagenomes and observed rates of enzymes from the GoM. Examples of the histograms of the observed rates describing four enzymes in the reference are shown. The solid red line represents the theoretical distribution. The dashed line is the observed rate for each enzyme in D18_MAX (blue) and A04_AAIW (green). The probability *P*_*gk*_ in Eq. 2 is the area under the red curve from minus infinity to the values indicated with these dashed lines.

The reference, as described in “Materials and Methods,” comprises a set of PDFs for those enzymes present in all 19 selected metagenomes. To obtain a random sampling and maximized variability of the metagenomes, we gathered samples located at different longitudes, latitudes, and depths. Figure 3 shows an example of the four types of PDFs of different enzymes: normal, log-normal, gamma, and Weibull. The enzymes described by gamma, log-normal and Weibull PDF are characterized by long tails. With these results, it is now simple to estimate, using Eq. 2, the probability of occurrence of observing a given rate (Figure 3). We used these theoretical distributions to analyze two metagenomes from the GoM. Instead of using the probability *P* directly, as described in “Materials and Methods,” it is convenient to transform it into a *z*-score using Eq. 3. These calculations can be easily performed with the appropriate software. We provide an example of the code used in R in Supplementary File 1. If the distribution is normal, the *z*-score can be calculated by hand with the usual formula. As is well known, the *z*-score in the center of the curve is zero, meaning that enzymes with values close to zero behave the same as in the reference. The *z*-scores to the right of the mean are positive, and the *z*-scores to the left of the mean are negative. Additionally, an important property of the *z*-score is that it can be used to calculate the percentage of the population below a given value. For this study, enzymes of the GoM presenting an EZS ≥ 2.0 have a rate higher than 97.72 of the reference observations. The equivalent *z*-score (EZS) above this region should be considered to have an atypical behavior; i.e., the enzymes with an EZS ≥ 2 are involved in metabolic functions overrepresented in the GoM communities. However, the enzymes located in the negative part of the curve, with an EZS ≤ −1, are also atypical but may be more relevant in the reference’s metagenomes. Finally, those enzymes in the interval between −1 > EZS ≤ 2 should be relevant for the communities represented in both the reference and the GoM metagenomes.

### Statistical Properties of the Reference Samples

As described in the methodology section, we calculated the PDF for each enzyme appearing in all 19 analyzed reference metagenomes. After the procedure was performed, 1,127 enzymes with a full EC description available were retained for posterior analysis. The statistical analysis showed that 49 (4%) enzymes had a gamma distribution, 257 (23%) had a Weibull distribution, 356 (32%) presented a log-normal distribution, and 465 (41%) showed a normal distribution (Supplementary Table 2). We then categorized the enzymes into their respective metabolic pathways, which placed 879 enzymes in at least one metabolic pathway (Supplementary Table 3), distributed as follows: gamma = 34 (4%), normal = 380 (43%), Weibull = 196 (22%), and log-normal = 269 (31%). According to this procedure, 40% of the enzymes showed a normal distribution, which can potentially be explained by different hypotheses, as explained in “Discussion.”

We then analyzed the behavior of enzymes essential for the maintenance of cellular integrity that should be present in every metagenome. Examples of such proteins include RNA polymerase subunits, aminoacyl-RNAt synthetase (aaRS), ribosomal proteins, and some metabolic enzymes that have been identified in representative organisms (Na et al., 2018). The KEGG Ortholog (KO) (Kanehisa et al., 2016) and Cluster of Orthologous Genes (COGs) databases (Tatusov et al., 2003; Galperin et al., 2015) include orthologous groups for most of these proteins, providing an initial view of the distribution of these enzymes in sequenced genomes. To evaluate the PDFs of essential enzymes found in the reference metagenomes, we searched for those with an EC number. The analysis revealed that among a group of 80 essential enzymes reported in the literature (Na et al., 2018), 16 were not present in the reference metagenomes, and 48 had a normal distribution. Fourteen enzymes presented a log-normal distribution, and two presented a Weibull distribution. From these enzymes, we observed that five enzymes of class 6.1.1.X (where X stands for any digit) presented non-normal PDFs, and all of them acted as aaRSs. As shown in Supplementary Table 3, three have a log-normal distribution, and two have a Weibull distribution behavior. To understand whether the total number of each aaRS distributed across a set of 4,852 bacterial genomes influences the observed PDF, we counted the orthologous genes in KO groups encoding aaRSs. Additionally, we grouped the aaRSs into their catalytic domain classes, where class I and class II aaRSs are unrelated (Woese et al., 2000; O’Donoghue and Luthey-Schulten, 2003). Supplementary Table 3 shows that none of these features may be responsible for the observed distributions since these enzymes present unique copies in the bacterial genomes.

In the next step, we explored whether the enzymes were equally distributed in the pathways, which was not the case, as shown in Supplementary Table 4. We observed poor preservation of the PDFs within the pathways, with between two and three enzymes being identified on average. We believe that this is an expected result since gene abundances tend to vary as a function of the relative abundance of the organisms constituting the sample. A remarkable case was that of the six enzymes involved in xylene metabolism, which displayed a Weibull distribution (Supplementary Table 4).

### Role of the Equivalent *z*-Score in the Identification of Metabolic Pathways

**FIGURE 4:**
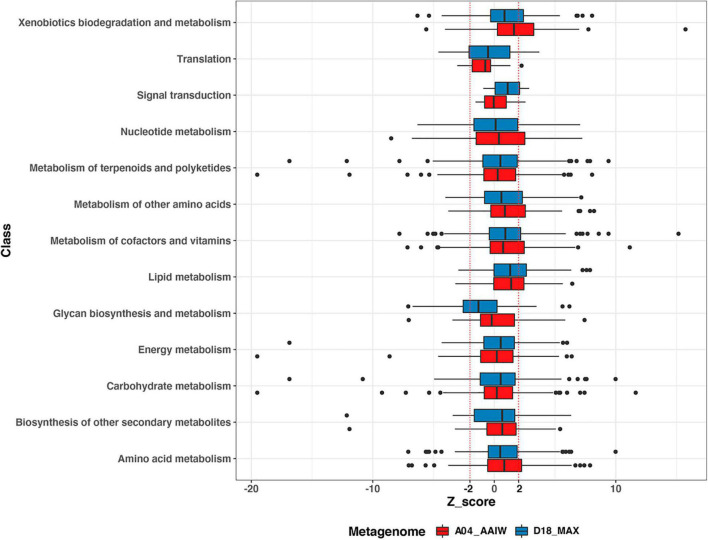
Enzyme distribution according to BRITE class. The distribution of the EZSs of the enzymes from A04_AAIW (red) and D18_MAX (blue) in KEGG BRITE classes is shown. EZSs (–2 and 2) are delimited by dotted red lines.

The 879 enzymes that were associated with a metabolic pathway and had an EZS were organized into the B functional classes stored in the KEGG BRITE database (Kanehisa, 2017; Supplementary Table 5), yielding thirteen classes, as shown in Figure 4. The enzymes from both metagenomes were classified into 11 metabolic classes, including four enzymes in the signal transduction category and 25 into the translation class containing the aaRs. A small number of enzymes appearing in both metagenomes catalyzed reactions related to infectious diseases in the following categories: viral, immune system (*n* = 2); environmental adaptation, endocrine system (*n* = 1); and drug resistance: antimicrobial (*n* = 1).

Among the boxplots shown in Figure 4, xenobiotic biodegradation stands out because in A04_AAIW, 24 enzymes of the 56 with significant EZS are included in the interval between the median and the third quartile (q3). The enzymes related to lipid metabolism in both metagenomes A04_AAIW (19 from 29) and D18_MAX (18 from 23) exhibit similar behavior. Some classes, such as nucleotide metabolism, metabolism of cofactors and vitamins, nucleotide metabolism in A04_AAIW, and the metabolism of cofactors and vitamins in D18_MAX, showed a lower proportion of significant enzymes between q3 and EZS ≥ 2 (Supplementary Table 6).

To evaluate the pathways defining the metabolic potential of both metagenomes, we took as a starting point the BRITE classes showing the highest proportion of enzymes with a significant EZS in q3 and beyond in the distribution.

### The Xenobiotic Class Includes Overrepresented Enzymes

**FIGURE 5:**
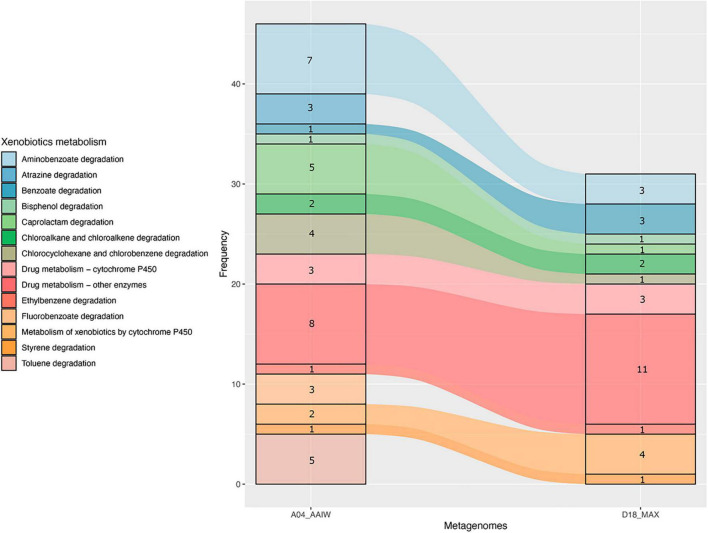
Pathways in the xenobiotics class. Counts of enzymes with EZS ≥ 2 found in pathways grouped in the xenobiotic KEGG BRITE class in the A04_AAIW and D18_MAX metagenomes.

As shown in Figure 4, a significant proportion of the enzymes catalyzing xenobiotic-related reactions presented an EZS ≥ 2. These enzymes, with are related to the following KEGG maps (as shown in Figure 5): aminobenzoate, atrazine, benzoate, bisphenol, caprolactam, chloroalkane, chlorocyclohexane and chlorobenzene degradation, drug metabolism – cytochrome P450, drug metabolism – other enzymes, ethylbenzene degradation, metabolism of xenobiotics by cytochrome P450, styrene degradation, and toluene degradation. Unlike A04_AAIW, the annotated enzymes from the D18_MAX metagenome in the xenobiotic class presented a lower count.

The results presented in Figure 5 show that the number of enzymes found in the KEGG xenobiotic maps with a EZS ≥ 2 were those of drug metabolism – other enzymes (A04_AAIW and D18_MAX), and aminobenzoate (A04_AAIW). Therefore, we proceeded to reconstruct a compound-reaction-enzyme network to inspect the number of consecutive steps leading to the inference of the pathways representing each metagenome. As described in detail in “Materials and Methods,” those enzymes with an EZS ≥ 2 were initially considered in the network reconstruction, followed by enrichment with enzymes in an interval between −1 ≥ EZS < 2. We assumed that the enzymes in the interval were equally represented in the reference metagenomes and the tested samples from the GoM. The networks representing each metagenome are shown in Supplementary Figures 1, 2.

From the network, it was evident that the pathways of drug metabolism (other enzymes) in A04_AAIW showed several connected reactions in which the enzymes presented a significant EZS (Supplementary Figure 1). Among these pathways, the enzymes involved in fluorouracil transformation stand out. In this pathway, the prodrugs capecitabine, tegafur, and carmofur, which are masked-form analogs of 5-fluorouracil (5-FU), are metabolized. These analogs are widely used to treat breast and gastrointestinal carcinomas (Uekama and Hirayama, 2008).

The results shown in Supplementary Table 5 for the metagenome show the enrichment of the carboxylesterase (EC: 3.1.1.1, EZS = 3.75583), which modifies these compounds in the liver of mammals (Wang et al., 2018). However, homologs of these enzymes can catalyze the production of analog compounds involved in bacterial metabolism, such as the enzyme EC 3.1.1.1, which has been described as an extracellular esterase and has been applied to determine marine bacterial metabolic activity and phytoplankton cell lysis (Barzkar et al., 2021). These extracellular enzymes have been reported to play a role in organic matter mineralization in the ocean (Barzkar et al., 2021). The drug metabolism-other enzymes map also comprises the enzymes thymidine phosphorylase (EC: 2.4.2.4, EZS = 3.61499) and a thymidine kinase (EC: 2.7.1.21, EZS = 5.02949) that incorporates thymidine in bacteria, including oceanic species (Jeffrey and Paul, 1990). Homologs of these enzymes modify other substrates in alternative pathways found in bacteria, in which thymidine phosphorylase catalyzes the conversion of thymine to thymidine in a reversible reaction. A recent study analyzing the functional metatranscriptomic enrichment of a marine oil spill in Bohai Bay in China showed that thymidine phosphorylase was one of the most upregulated enzymes (Song et al., 2021). The subsequent steps transforming thymidine to deoxythymidine monophosphate (dTMP) and dTMP to deoxythymidine diphosphate (TDP), catalyzed by 5′-nucleotidase (EC: 3.1.3.5, EZS = 3.2916) and dTMP kinase (EC: 2.7.4.9, EZS = 4.28755), respectively, were also detected at a significant level; however, the final step in which dTDP is transformed to deoxythymidine triphosphate (dTTP), catalyzed by the nucleoside-diphosphate kinase EC 2.7.4.6, was underrepresented (EZS = −1.14672). Remarkably, within this pathway, 5′-nucleotidase is a key enzyme involved in aquatic phosphorus regeneration, even in the open ocean (Ammerman and Azam, 1985). Our analysis showed that alkaline phosphatase is a bacterial membrane protein encoded by the *phoA* gene (Inouye et al., 1981) that has been hypothesized to be directly related to phosphorus deficiency within bacterial cells (Wanner, 1996; Benitez-Nelson, 2000), presented an EZS = 1.52342 in A04_AAIW and an EZS = 2.01831 in D18_MAX, suggesting that the microbial community in the MAX zone must have the potential to contend with inorganic phosphate (Pi) stress.

### Enzymes Involved in Petroleum Hydrocarbon Degradation Are Significantly Represented in the Perdido Fold Belt and Campeche Knolls in the Gulf of Mexico

**FIGURE 6:**
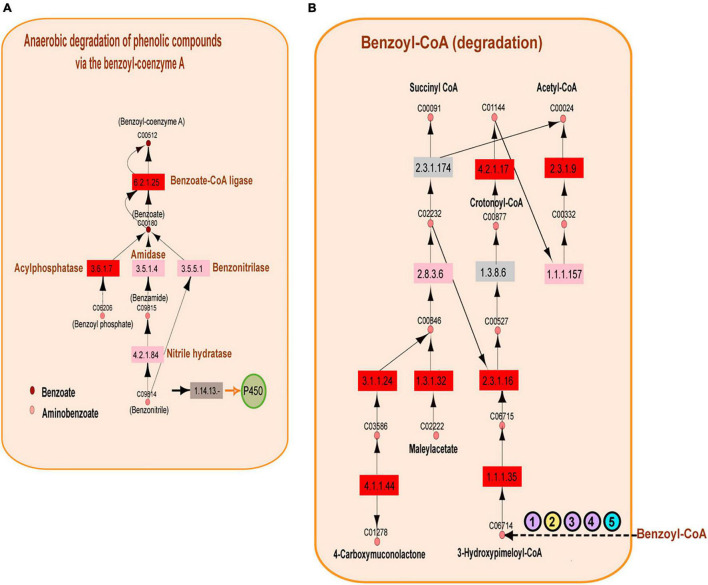
Benzoyl-coenzyme A metabolism. **(A)** Reconstruction of the anaerobic degradation of phenolic compounds *via* the benzoyl-coenzyme A pathway. Significantly represented enzymes are shown in red, the enzymes represented equally in the reference and in the A04_AAIW metagenome are shown in pink, and the enzymes that are better represented in the reference metagenomes are shown in gray. The key enzymes related to benzoyl-coenzyme A are shown blue. Pink circles represent the compounds found in the KEGG aminobenzoate pathway, and brown circles represent those of the benzoate pathway. **(B)** Metabolic network reconstruction of the anaerobic conversion of benzoyl-coenzyme A. The significant enzymes are shown in red; the enzymes represented equally in the reference and the A04_AAIW metagenome are shown in pink, and the enzymes that are better represented in the reference metagenomes are shown in gray. A group of enzymes annotated in the reference and A04_AAIW (purple circles) with EC numbers “. -” are presented. One enzyme annotated only in the reference yellow circle and another annotated only in the Gulf metagenome (blue circle) are also shown (the EC list is included in Supplementary Table 7).

The above observations naturally showed an overrepresentation of enzymes involved in petroleum degradation, consistent with the geographical origin of samples extracted from the GoM. The results presented in Figure 5 show the enrichment of enzymes catalyzing aromatic hydrocarbon degradation. In the A04_AAIW metagenome, extracted from a depth of 1,000 m, we detected 46 enzymes with significant EZSs that catalyze reactions included in 14 KEGG pathway maps. The KEGG maps of aminobenzoate, caprolactam, and toluene degradation were the most populated. We then performed a deeper inspection of these KEGG maps to identify two features: (1) the presence of key enzymes in the pathways with significant EZSs and (2) consecutive reactions with a significant EZS. From this analysis, two consecutive reactions caught our attention; the first transforms benzoyl-phosphate to benzoate, and the second transforms benzoate to benzoyl-CoA. These reactions are catalyzed by the enzymes acylphosphatase (EC: 3.6.1.7, EZS = 5.06032) and benzoate-CoA ligase (EC: 6.2.1.25, EZS = 5.74931) (as shown in Figure 6A), both of which are key enzymes in anaerobic benzoyl-CoA degradation. To date, the anaerobic degradation of monoaromatic hydrocarbons, such as benzoate, phenol, toluene, and ethylbenzene, has been indicated to proceed *via* a pathway involving benzoyl-CoA (Boll, 2005; Meckenstock and Mouttaki, 2011).

The following steps in the conversion of benzoyl-CoA to acetyl or succinyl-CoA can proceed *via* several different pathways that are well described in *Thauera aromatica*, *Azoarcus* sp. *CIB*, and *Geobacter metallireducens*, in which benzoyl-CoA is reduced to formcyclohexa-1,5-diene-1-carbonyl-CoA by a benzoyl-CoA reductase (Porter and Young, 2014). The reduction of benzoyl-CoA to cyclohexa-1,5-diene-1-carbonyl-CoA also occurs in *Rhodopseudomonas palustris*, and a common hydrolysis step occurs in all of these species (Porter and Young, 2014). After this step, the conversion of cyclohexa-1,5-diene-1-carbonyl-CoA continues *via* different pathways. In our study, we did not identify the pathways found in *T. aromatica*, *Azoarcus* sp. *CIB*, or *G. metallireducens*, but our statistical analysis yielded several enzymes involved in benzoyl-CoA conversion in *R. palustris*. The first notable result showed that enzymes transforming benzoyl-CoA to 3-hydroxy-pimeloyl-CoA were not detected; however, as shown in Figure 6B, the enzymes that subsequently transform 3-hydroxy-pimeloyl-CoA to 3-keto-pimeloyl-CoA (3-hydroxyacyl-CoA dehydrogenase; EC: 1.1.1.35, EZS = 3.27996) and 3-keto-pimeloyl-CoA to glutaryl-CoA (acetyl-CoA acyltransferase; EC: 2.3.1.16, EZS = 2.44283) presented significant EZSs. The enzyme acetyl-CoA acyltransferase (EC: 1.3.8.6), which transforms glutaryl-CoA to crotonyl-CoA, was also missing. Nevertheless, enoyl-CoA hydratase (EC: 4.2.1.17, EZS = 4.00025), which catalyzes the transformation of crotonyl-CoA to (S)-3-hydroxybutanoyl-CoA; 3-hydroxybutyryl-CoA dehydrogenase (EC: 1.1.1.157), which catalyzes the transformation of (S)-3-hydroxybutanoyl-CoA to acetoacetyl-CoA catalyzed; and acetyl-CoA C-acetyltransferase (EC: 2.3.1.9, EZS = 2.44034), which is responsible for the catalysis of acetoacetyl-CoA to acetyl-CoA, were identified as significant enzymes by this method. The analysis of the benzoate KEGG map showed that several enzymes involved in the transformation of benzoyl-CoA to 3-hydroxy-pimeloyl-CoA do not have a full EC number description. This was the case for the benzoyl-CoA reductase subunit BamB (EC: 1.3.-.-), cyclohex-1-ene-1-carboxyl-CoA (EC: 4.2.1.-), hydratase, 2-hydroxycyclohexanecarboxyl-CoA dehydrogenase (EC: 1.1.1.-), and 2-ketocyclohexanecarboxyl-CoA hydrolase (EC: 3.1.2.-), which were annotated by MGRast in both the A04_AAIW metagenome and in the references (Figure 6B). Enzymes of this nomenclature were dismissed in the initial analysis. In 2018, they were discarded from the records of protein and metabolic databases, including KEGG, since they represent incomplete enzyme-catalyzed reactions.^4^ On the other hand, benzoyl-CoA reductase subunit C (EC: 1.3.7.8) was annotated in our metagenome but not in the reference samples, and cyclohexane-1-ene-1-carbonyl-CoA dehydrogenase (EC: 1.3.8.10) was annotated in the reference but not in our metagenomes. Finally, the enzyme (EC: 1.3.62.80) pimeloyl-CoA dehydrogenase was not detected in the reference or in A04_AAIW. We will later discuss the preprocessing and postprocessing methodologies that may affect enzyme identification in metagenomic samples.

Additionally, in the aminobenzoate KEGG map, we found that the enzyme phenol hydroxylase (EC: 1.14.13.7, EZS = 2.18523) involved in the elimination of phenolic compounds was significantly represented in A04_AAIW (Supplementary Table 5). In phenol degradation, phenol hydroxylase monohydroxylates the aromatic ring in the adjacent carbon of a hydroxyl group, resulting in catechol, which is in turn cleaved by either *ortho*- or *meta*-cleavage pathways (Silva et al., 2013). These pathways have been described in the genus *Halomonas*, which is able to degrade phenol as a sole source of carbon and energy, as observed in a *Halomonas* sp. strain isolated from the Great Salt Lake (Le Borgne et al., 2008). In our previous study, we analyzed the bacterial community of A04_AAIW; we observed that the abundance of the genus *Halomonas* increased substantially (1–48%) (Raggi et al., 2020). From the posterior conversion of catechol, we found that the ortho-cleavage pathway included significant EZSs of muconate cycloisomerase (EC: 5.5.1.1, EZS = 2.3406), 3-oxoadipate enol-lactonase (EC: 3.1.1.24, EZS = 6.4463), and the acetyl-CoA acyltransferase (EC: 2.3.1.16, EZS = 2.44283), which were significantly represented. Two other enzymes, the 3-oxoadipate CoA-transferase alpha subunit (EC: 2.8.3.6 EZS = 0.0846268) and 3-oxoadipyl-CoA thiolase (EC: 2.3.1.174, EZS = −0.373101), had EZSs located within the interval of −2 ≥ EZS < 2. As previously mentioned, we considered values in the interval of −1 ≥ EZS < 2 to correspond to enzymes represented in both the reference and the queried metagenomes.

**FIGURE 7:**
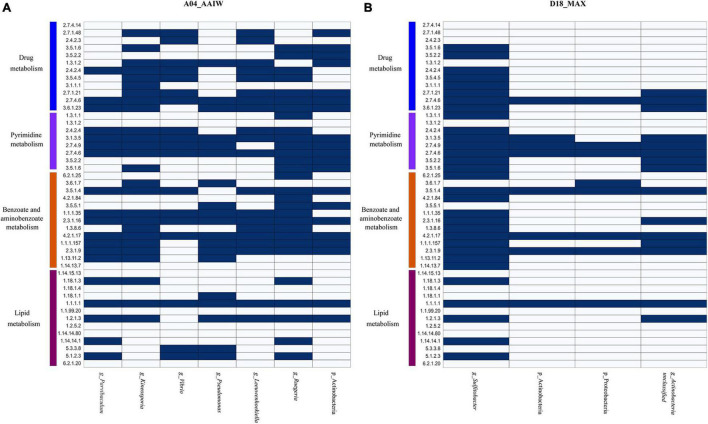
Metabolisms described in the binned metagenomes **(A)** A04_AAIW and **(B)** D18_MAX. The heat maps depict the presence (blue) and absence (white) of enzymes involved in drug metabolism, other enzymes, pyrimidine, benzoate, aminobenzoate, and lipid metabolisms. The explored pathways represent the metabolic profile found in metagenomic bins, which metabolically and taxonomically describe the nature of the samples.

Finally, to identify the taxonomy that may be involved in oil degradation and some of the above-described pathways, we binned and classified the genomes from the metagenomes. The taxonomy found in A04_AAIW, as shown in Figure 7A, reveals and matches the capability of hydrocarbon degradation, represented by *Parvibaculum lavamentivorans*, which is usually detected in habitats contaminated with oil, diesel, and other compounds (Schleheck et al., 2011). We also identified in A04_AAIW, *Pseudomonas aestusnigri*, isolated from an oil spill (Sánchez et al., 2014), and organisms within the genera *Kineosporia* that are also capable of hydrocarbon degradation (Gontikaki et al., 2018). In the D18_MAX metagenome, we identified *Sulfitobacter* sp. DFL_14, an oil degrader (Nuñal et al., 2017), and *Actinobacteria*_unclassified_SGB36491 that have enzymes related to aminobenzoate and benzoate degradation (Figure 7B).

### Lipid Metabolism Is Related to Petroleum Degradation

Lipids are hydrocarbons that are soluble in non-polar solvents (Pond, 2009). They are also carriers of fat-soluble vitamins, carotenoids, and organic contaminants, the last of which are the main drivers of pollutant bioaccumulation in marine ecosystems (Parrish, 2013). Both A04_AAIW and D18_MAX showed enrichment in enzymes related to lipid metabolism. Lipid enzymes are involved in pathways such as the biosynthesis and degradation of essential fatty acids, glycolipids, and glycerophospholipids. These pathways include hydrocarbons in the form of alkanes that are modified *via* terminal oxidation to fatty acids. In A04_AAIW (Supplementary Table 5), we found enzymes related to alkane degradation, such as alkane 1-monooxygenase (EC:1.14.15.3, EZS = 6.41577), which was one of the most enriched enzymes in the dataset. Within this pathway, we found that aldehyde dehydrogenase (NAD+) was significantly enriched (EC: 1.2.1.3, EZS = 2.19467). Other enzymes, such as alcohol dehydrogenase (EC: 1.1.1.1), ferredoxin–NAD+ reductase (EC: 1.18.1.3) and rubredoxin–NAD+ reductase (EC: 1.18.1.1), were equally represented in both the reference and A04_AAIW samples, indicating that the pathway may be present in some species in the sample. It has been reported that the genus *Alcanivorax* is abundant in alkane-polluted ocean waters (Li et al., 2019), and in a recent study, we reported the presence of genes involved in alkane degradation in *Pseudomonas aeruginosa* strain GOM1 isolated from the water column (Muriel-Millán et al., 2019). Moreover, we have previously reported the particular enrichment of Alcanivorax (23%) at this site (Raggi et al., 2020). A set of enzymes involved in fatty acid oxidation that were significantly detected in A04_AAIW included dodecenoyl-CoA isomerase (EC: 5.3.3.8, EZS = 2.19995), which participates in the beta-oxidation of fatty acids with double bonds at an odd position, and 3-hydroxybutyryl-CoA epimerase (EC: 5.1.2.3, EZS = 2.67964), which is also involved in fatty acid beta-oxidation. The long-chain fatty acid–[acyl carrier protein] ligase (EC: 6.2.1.20, EZS = 0.403923) showed a score suggesting that the enzyme is equally represented in the A04_AAIW and reference metagenomes. Alkane degradation enzymes were poorly enriched in D18_MAX, except for aldehyde dehydrogenase (EC: 1.2.1.3, EZS = 2.19467). Five of the enzymes in the pathway were represented both in the reference and in our metagenome, which suggests that there are organisms in all of these samples that are capable of transforming alkanes. Searching into the binned genomes (Figures 7A,B), we found the ferredoxin–NAD+ reductase (EC: 1.18.1.3), alcohol dehydrogenase (EC: 1.1.1.1), and aldehyde dehydrogenase (NAD+), represented in *P. lavamentivorans* (A04_AAIW) and *Sulfitobacter* sp. DFL_14 (D18_MAX), both reported as oil degraders as previously commented.

### Networks Representing the Metabolic Potential

The examples presented in the previous section showed pathway reconstructions considering the BRITE classes with wider enrichment to be those related to xenobiotic and lipid metabolism. However, as shown in Figure 4, other classes included enzymes implicated in different metabolic pathways. Therefore, to obtain a global view of the metabolic potential detected in both metagenomes, we assessed groups of enzymes with −1 ≥ EZSs ≥ 2, which were used to construct the metabolic network of A04_AAIW and D18_MAX (Supplementary Figures 1, 2). As explained in detail in “Materials and Methods,” the network was built using the substrates and products catalyzed by the selected enzymes.

**FIGURE 8:**
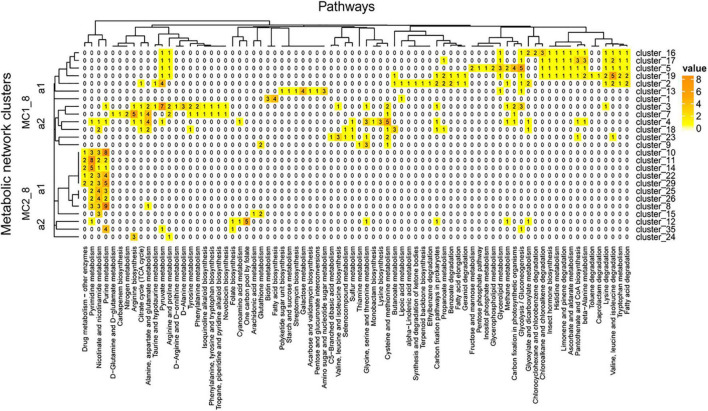
A04_AAIW metagenome metabolic network cluster. We present the clustering analysis of the A04_AAIW metabolic networks. The analysis represents the total number of intersecting ECs between each group and the KEGG pathways employed for global network construction.

The network was grouped into 35 clusters in A04_AAIW and 51 in D18_MAX, from which we extracted those clusters included more than 4 enzymes. These clusters were regrouped to evaluate the interactions within the network (Supplementary Table 8). The clusters derived from sample A04_AAIW are represented in Figure 8, in which we observed two major clusters. Major cluster MC1_8 was subdivided into two subclusters, MC1_8_a1 and MC2_8_a2. MC1_8_a1 contains clusters 1, 2, 5, 13, 16, 17, and 19, among which clusters 1, 2, and 13 did not share any reactions catalyzed by the same group of enzymes. The most populated clusters (which we defined as those having at least 3 enzymes associated with a metabolic pathway), were related to the catalysis of reactions involved in biotin metabolism and fatty acid biosynthesis (cluster_3); galactose metabolism, amino sugar, and nucleotide sugar metabolism (cluster_13); and pyruvate metabolism (cluster_2).

In clusters 5, 16, 17, and 19, aldehyde dehydrogenase (NAD+) (EC: 1.2.1.3, EZS = 2.24583) catalyzes reactions of the fatty acid degradation pathway, valine, leucine, and isoleucine degradation, tryptophan metabolism, lysine degradation, β-alanine metabolism, pantothenate and CoA biosynthesis, limonene and pinene degradation, ascorbate and aldarate metabolism, and glycolysis/gluconeogenesis, among others. This enzyme has been characterized in marine bacteria such as *Halomonas salina* strain AS11, which produces high levels of aldehyde dehydrogenase (Sripo et al., 2002) that may contribute to the biotransformation of a large number of drugs and other xenobiotics; these reactions generate aldehydes as intermediates, many of which have significant biological effects, including cytotoxicity, mutagenicity, genotoxicity, and carcinogenicity, and they have also been identified as a product of the shrimp aquaculture industry (Sripo et al., 2002).

MC1_8_a2 contains clusters 9 and 23, responsible for catalyzing reactions involved in amino acid metabolism; it shows no related enzymes, and a larger subcluster includes clusters 3, 4, 7, and 14. Clusters 23 and 18 contain cystathionine gamma-synthase (EC: 2.5.1.48, EZS = 1.53123), which is shared by the reference and this metagenome. Cystathionine gamma-synthase catalyzes reactions involved in cysteine and methionine metabolism, seleno compound metabolism, and sulfur metabolism. Cluster 23 also shares with cluster 3 threonine ammonia-lyase (EC: 4.3.1.19, EZS = 3.46455), which was significantly detected in our metagenome. This enzyme is a D-amino acid lyase that, similar to other D-amino acid lyases, is a key enzyme involved in the use of D-amino acids as a nitrogen source integrating DOM in marine environments (Yu et al., 2020).

Major cluster MC2_8_a1 contains 12 groups, five of which are interconnected (clusters 8, 10, 22, 25, and 29) by purine nucleosidase (EC: 3.2.2.1, EZS = 1.036), with EZSs equally represented in the reference and the metagenome. This enzyme is involved in purine metabolism and nicotinate and nicotinamide metabolism. Clusters 10, 11, 14, 22, 25, 26, 29, and 10 share a 5′-nucleotidase (EC: 3.1.3.5, EZS = 3.2916) and purine nucleoside phosphorylase (EC: 2.4.2.1, ESZ = 3.19126), both of which have significant EZSs and catalyze reactions involved in purine metabolism, pyrimidine metabolism, nicotinate and nicotinamide metabolism, and the biosynthesis of secondary metabolites. In a previous section, we discussed the relevance of 5′-nucleotidase in the phosphate cycle. Clusters 11 and 14 share thymidine phosphorylase (EC: 2.4.2.4) and thymidine kinase (EC: 2.7.1.21), both of which were identified at significant levels and catalyze reactions involved in pyrimidine metabolism and drug metabolism. Clusters 10, 14, 22, and 29 contain enzymes related to drug metabolism – other enzymes, such as hypoxanthine-guanine phosphoribosyltransferase (PRTase) (EC: 2.4.2.8, EZS = 5.74335), whose activity is also associated with the purine salvage pathway that generates adenine and guanine ribonucleotides and deoxynucleotides from hypoxanthine. This enzyme shows broad specificity and is universally distributed in the three cellular domains (Armenta-Medina et al., 2014).

Other pathways involved in arginine metabolism and the one-carbon pool associated with folate were identified in MC2_8_a2, which included some enzymes related to nucleotide metabolism. In particular, the one-carbon pool involved in folate metabolism was associated with a cluster of five enzymes, three of which were detected at significant levels: dihydrofolate reductase (DHFR) (EC: 1.5.1.3, EZS = 4.87773), formyltetrahydrofolate deformylase (EC: 3.5.1.10, EZS = 2.91206), and methionyl-tRNA formyltransferase (EC: 2.1.2.9, EZS = 2.24395). DHFR catalyzes the reduction of dihydrofolate (DHF) to tetrahydrofolate (THF); this is an indispensable enzyme for all organisms because of its role in the biosynthesis of purine nucleotides and some amino acids (Ohmae et al., 2015). This enzyme, as well as others that were well represented in our study such as alpha-glucosidase (Shirai et al., 2008) (EC: 3.2.1.20, EZS = 2.64074) and aspartate carbamoyltransferase (De Vos et al., 2007) (EC: 2.1.3.2, EZS = 2.24583), have been cataloged as deep-sea enzymes. Another deep-sea enzyme is 3-isopropyl malate dehydrogenase (Kato et al., 1998) (EC: 1.1.1.85 = 0.847829), which was equally represented in the GoM metagenome and the reference samples.

**FIGURE 9:**
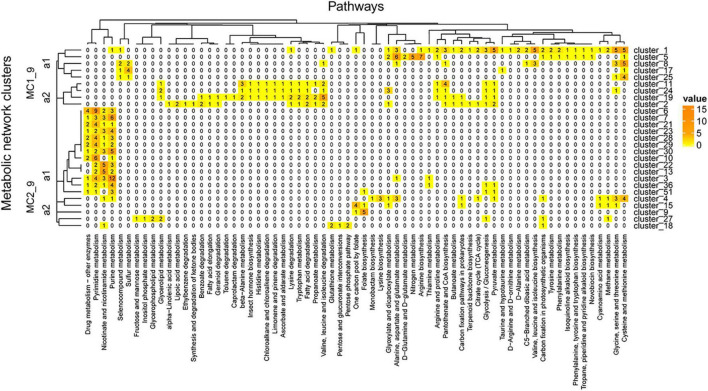
D18_MAX metagenome metabolic network cluster. We present the clustering analysis of the D18_MAX metabolic networks. The analysis represents the total number of intersecting ECs between each group and the KEGG pathways employed for global network construction.

The clusters in Figure 9 showed pathways enriched in the zone of maximum fluorescence (D18_MAX). In this metagenome, we identified 27 clusters, some of which were enriched at this depth: drug metabolism – other enzymes, pyrimidine and purine metabolism and nicotinate and nicotinamide metabolism, among others. On the other hand, pathways involved in the xenobiotics class were more represented in the network than at the A04_AAIW depth; these pathways included the ethylbenzene, geraniol, caprolactam, toluene, chloroalkane and chloroalkane, and limonene degradation pathways. Similarly, as shown in Figure 8, the tree can be divided into two major clusters (MC1_9 and MC2_9).

MC1_9_a1 includes clusters 1, 5, 8, 17, and 25. Cluster 1, the largest of these clusters, contains enzymes catalyzing reactions in other clusters of the same group. The shared enzymes in this cluster are equally represented in both our metagenome and the reference. The pathways with more than 3 enzymes include several amino acid biosynthesis and degradation pathways, such as glycine, serine, and threonine metabolism; cysteine and methionine metabolism; arginine biosynthesis; and alanine, aspartate, and glutamate metabolism. Nitrogen metabolism involves five enzymes catalyzing steps including the transformation of nitrile to ammonia (NH3+) (mediated by glutaminase; EC 3.5.1.2, EZS = 4.26525), which is then transformed into L-glutamine (mediated by glutamine synthetase; EC 6.3.1.2, EZS = 0.98244) and further into L-glutamate [mediated by three enzymes, glutamate synthase (NADPH) large chain (EC: 1.4.1.13, EZS = 0.255537), glutamate synthase (NADH) (EC: 1.4.1.14, EZS = −0.495233), and glutamate synthase (ferredoxin) (EC: 1.4.7.1, EZS = −0.823102)]. An alternative pathway with the significant role of directly transforming ammonia into L-glutamate, mediated by glutamate dehydrogenase [NAD(P)+] (EC: 1.4.1.3, EZS = 5.65565), was also identified. The end product of this reaction enters D-glutamine and D-glutamate metabolism, in which glutamate is reversibly transformed into two oxoglutarate by glutamate dehydrogenase [NAD(P)+]. In turn, two oxoglutarate is taken up by the citrate cycle or is transformed into D-glutamate by glutamate racemase (EC: 5.1.1.3, EZS = 2.30112). D-Glutamate is subsequently transformed into UDP-*N*-acetylmuramoyl-L-alanyl-D-glutamate by UDP-*N*-acetylmuramoylalanine-D-glutamate ligase (EC: 6.3.2.9, EZS = 6.18275), and the next product enters peptidoglycan biosynthesis. All of the enzymes of this pathway have significant EZSs. The abovementioned findings are important since although D-amino acids are minor components of living organisms, they occur in a wide range of natural environments, such as soils (Vranova et al., 2012; Naganuma et al., 2018), rivers, and marine systems (Wu et al., 2007; Naganuma et al., 2018), among others.

MC1_9_a1 includes four populated pathways: β-alanine metabolism; valine, leucine, and isoleucine degradation; glyoxylate and dicarboxylate metabolism; and cysteine and methionine metabolism. Clusters 11, 19, and 24 contain aldehyde dehydrogenase, which participates in alkane degradation as previously mentioned. This versatile enzyme in this cluster also participates in the production of β-alanine. From this pathway, we identified all of the steps in the bidirectional conversion of uracil into β-alanine. The enzymes participating in this pathway are dihydropyrimidine dehydrogenase (NAD+) subunit PreT (EC: 1.3.1.1, EZS = 2.03861) and dihydropyrimidine dehydrogenase (NADP+) (EC: 1.3.1.2, EZS = 1.38085), which catalyzes the transformation of uracil into 5,6-dihydrouracil, followed by the reaction converting this compound into *N*-carbamoyl-β-alanine, catalyzed by dihydropyrimidinase (EC: 3.5.2.2, EZS = 0.631778). The last reaction is the conversion to β-alanine by the enzyme beta-ureidopropionase (EC: 3.5.1.6, EZS = −0.734533). Similar results have shown that the non-protein amino acids β-alanine and γ-aminobutyric acid can be found as dissolved free amino acids in marine water (Dawson, 1986). The sequence of reactions found in our study suggests the presence of microorganisms capable of decomposing uracil into β-alanine. Finally, MC1_9_a2 includes enzymes that catalyze reactions involved in drug metabolism - other enzymes, pyrimidine metabolism, nicotinate and nicotinamide metabolism, and purine metabolism. These pathways were also found in A04_AAIW, but a greater proportion of the enzymes were represented in D18_MAX, with a maximum of 12 enzymes, versus a maximum of 9 in A04_AAIW. Enzymes representative of cluster 3 were the most abundant (*n* = 12), all of which participated in reactions involved in purine metabolism.

## Discussion

### Reference Metagenomes as Tools for the Identification of Metabolic Potential

Extracting meaningful biological information from metagenomes is a serious bioinformatics challenge. A considerable number of tools have been employed to identify useful information that can help to understand the taxonomic and genomic composition of several biomes (De Anda et al., 2017; Nurk et al., 2017; Escobar-Zepeda et al., 2018; Dong and Strous, 2019; Paczian et al., 2019). Consequently, the need for a new method that takes into consideration all the information of the enzymes in a group of metagenomes, unlike previously described approaches that depict metabolic pathways only based on a group of marker genes, led us to propose a novel statistical method that considers the distribution of enzymes derived from the annotation of metagenomic samples as a fundamental element of the analysis. The logic underlying this approach is that each enzyme in a group of metagenomes should have a particular distribution and that the EZS can indicate whether an enzyme in a different metagenome is distinct from those observed in the reference distribution. As shown by our results, 41% of the enzymes had a normal distribution. We interpreted this as better preservation of these enzymes in the metagenomes. This possibility was evaluated by observing the distribution of essential enzymes, which showed that 77% of them presented a normal, consistent with our expectation that these enzymes would be present in all organisms of the community.

However, as shown in the results, some enzymes, such as some of the aaRSs, are non-normally distributed. Moreover, the log-normal and Weibull observed distributions have long tails, which are characteristic of these kinds of distributions. The meaning of this behavior is difficult to explain; however, we know that these enzymes are not well preserved in known species, and several of them have been tested only in representative organisms. It is possible that the organisms with these aaRSs are not heavily represented in the selected samples. This lack of preservation of certain aaRSs has been shown for some archaeal, bacterial, and organellar genomes that instead employ indirect aminoacylation pathways (O’Donoghue and Luthey-Schulten, 2003), which may explain the observed behavior. Other explanations may be related to the impossibility of recovering genes of low-abundance organisms, or the genes encoding these enzymes may be in chromosomal regions that are difficult to sequence. On the other hand, the lack of coverage in the assembly could be another problem impacting the more homogeneous annotation of these enzymes.

As previously mentioned, identifying valuable information in metagenome samples is challenging since the methodological decisions made concerning trimming, assembly, and annotation can impact the results and affect the tested distributions, especially for those genes that are less widely distributed in the samples. However, in the analysis performed herein, we attempted to consider methodological and biological variation by evaluating the distribution of each enzyme.

Another factor affecting the PDFs of enzymes is the kind of metagenome selected to construct the reference dataset. The approach applied herein first considered samples collected worldwide, representing distinct depths and conditions, which were compared with the GoM samples under the assumption that they should differ, at least concerning the presence of hydrocarbons. However, the environmental conditions were poorly described in several metagenomic studies, even when physicochemical parameters were included. Therefore, the selection of reference samples could be challenging. In these cases, the analysis encountered reference conditions presenting small variations compared with our metagenomes. As a result, we found similarities between the samples, leading to poor enzyme identification, but we hope to identify some enzymes defining the tested conditions. Additionally, the databases used for annotation will introduce bias. In this work, we employed MG-RAST, which uses several databases to perform this task. Nonetheless, the annotations are incomplete.

It may be possible to improve our statistical method by modifying the selection of the reference samples. This can be done by performing sampling at several points at the same depth, longitude, and latitude and probably in the same season, considering the impact of this last parameter on the biophysiochemical composition of ocean samples, which we think will highlight the uniqueness of certain enzymes in the tested metagenomes. Moreover, by selecting the reference in this way, we expect to increase the detection of enzymes in the reference since we expect all of them to be preserved in the metagenomes; however, this is also a disadvantage since not all enzymes are represented in all metagenomes. Nevertheless, we wanted to be strict concerning this point. We also expect the enzymes to be more normally distributed in these new references, considering that the samples may be more similar.

### Identification of Overrepresented Pathways

The boxplots showing the distribution of EZS in Figure 4 were the initial approximations used for the selection of major classes with a high proportion of atypical enzymes. The xenobiotic pathways in the A04_AAIW metagenome showed this property. This is a meaningful result knowing that our samples were collected in the GoM, which is frequently disrupted by both natural oil release (De Beukelaer et al., 2003; MacDonald et al., 2015) and oil spills (Mason et al., 2012; Liu and Liu, 2013; Looper et al., 2013). Moreover, the natural identification of the anaerobic degradation of aromatic hydrocarbons was meaningful at this depth, proving that our method is a powerful tool for discovering well-represented enzymes that correlate well with the physicochemical composition of the environment without any previous preselection.

The approach also naturally highlighted other enzymes related to petroleum biodegradation and those involved in lipid and amino acid metabolism in both metagenomes, in accord with previous studies that have demonstrated that “crude oil pollution constitutes a temporary condition of carbon excess coupled to limited availability of nitrogen that prompts marine oil-degrading bacteria to accumulate storage compounds” (Manilla-Pérez et al., 2010) such as lipids. In aquatic ecosystems, lipids provide the densest form of energy, yielding at least two-thirds more energy per gram than proteins or carbohydrates, and they are therefore significant molecules in trophic chains (Parrish, 2013). As noted above, lipids also function as solvent or absorbent carriers of fat-soluble vitamins, carotenoids, and organic contaminants, the last of which are the main drivers of pollutant bioaccumulation in marine ecosystems (Parrish, 2013). Additionally, certain essential fatty acids and sterols are important drivers of ecosystem health and stability (Arts et al., 2001).

Notably, the results related to the presence of enzymes involved in oil degradation are consistent with previous works of our own and other groups that investigated the Mexican exclusive economic zone and identified oil-degrading microorganisms derived from 16S amplicon analysis (Fernanda Sánchez-Soto Jiménez et al., 2018; Godoy-Lozano et al., 2018; Hernández-López et al., 2019; Moreno-Ulloa et al., 2019; Raggi et al., 2020; Ramírez et al., 2020); isolated fractions related to petroleum (Moreno-Ulloa et al., 2019); isolated consortia and species capable of degrading petroleum under aerobic and anaerobic conditions (Muriel-Millán et al., 2019; Curiel-Maciel et al., 2021; Rosas-Díaz et al., 2021); and characterized petroleum-degrading enzymes (Rodríguez-Salazar et al., 2020), even in regions with no evidence of anthropogenic perturbation. A recent review illustrated the distribution of bacteria in the water column and sediments of the Mexican exclusive economic zone of the GoM (Rodríguez-Salazar et al., 2021). The identification of all samples amplified using the V3–V4 16S rRNA gene variable regions showed that members of oil-degrading genera were distributed in the water column throughout the region extending from Perdido (Tampico) to Campeche Bay.

In this study, the inspection of BRITE classes provided a general view of the global metabolism represented in the GoM metagenomes. Nevertheless, for the definition of a more detailed metabolic profile, network reconstruction is necessary. The reconstructed network revealed the interconnection of compounds whose production was catalyzed by the identified enzymes. This reconstruction considered not only the enzymes with a significant EZS ≥ 2 but also the enzymes in the interval between −1 and 2. As explained above, the EZSs located in the interval corresponded to enzymes that were equally represented in the reference and in the GoM metagenomes.

Network reconstruction led to the identification of several enzymes functioning in more than one reaction. These reactions were components of pathways that may not be in accord with the environmental conditions evaluated. Therefore, an important step of manual curation is always necessary to establish a more consistent pathway reconstruction. In our work, this was the case for the detected reactions in the drug metabolism – other enzymes category, composed of enzymes participating in the fluorouracil pathway, for which analogs are widely used to treat breast and gastrointestinal carcinomas (Uekama and Hirayama, 2008). The anticancer effects of these drugs in mammals are exerted through the inhibition of thymidylate synthase and the misincorporation of its metabolites into RNA and DNA in place of uracil or thymine. A deep inspection showed that a better interpretation of our observations was related to pyrimidine metabolism, which is closely related to the phosphorus cycle in oceans. However, it is worth noting that 5-FU derivatives were isolated from the marine sponge *Phakellia fusca* in another study (Xu et al., 2003), which is significant because fluorine-containing organic compounds are rare in nature; nevertheless, the presence of this type of metabolism in microorganisms has not been reported.

The analysis of pyrimidine metabolism provided an excellent example of the identification of key enzymes, such as the 5′ nucleotidase involved in aquatic phosphorus regeneration and the alkaline phosphatase modulating phosphorus deficiency within bacterial cells. The 5′nucleotidase was first identified in both marine and freshwater environments on the cell surface of bacterial plankton and was shown to rapidly hydrolyze 5′ nucleotides regenerating Pi (orthophosphate) (Bjorkman and Karl, 1994). This enzyme is different from alkaline phosphatase (EC: 3.1.3.1), another bacterial membrane protein encoded by the *phoA* gene (Inouye et al., 1981) that has been hypothesized to be directly related to phosphorus deficiency within bacterial cells (Wanner, 1996; Benitez-Nelson, 2000). Moreover, under laboratory conditions, the inhibition of the end product of alkaline phosphatase by phosphate demonstrated the relationship between low Pi levels and alkaline phosphatase activity (Mahaffey et al., 2014). The same study showed that the activity of alkaline phosphatase clearly impacted the concentration of dissolved organic phosphorus (DOP), which was suggested in a recent revision to potentially support a large fraction of the P demand in the microbial community (Duhamel et al., 2021). However, studies in the open ocean show that alkaline phosphatase activity occurs in a range of concentrations that suggest constitutive or inducible activity (Mahaffey et al., 2014).

Among these enzymes, we identified key enzymes of the benzoyl-CoA pathway and some deep-sea enzyme markers. Furthermore, the identification of the β-alanine pathway in D18_MAX was also consistent with reports showing that in the marine water column, these non-protein amino acids comprise a small fraction (<4%) of the total amino acid pool, which may be released during phytoplankton decomposition (Nguyen and Harvey, 1997) and then deposited in marine sediments, where they are more abundant (Schroeder, 1975).

**FIGURE 10:**
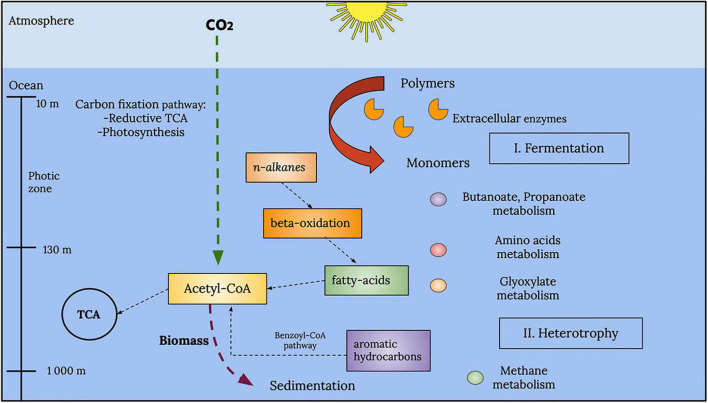
Global representation of the metabolic processes in the analyzed GoM samples. A series of fermentation processes, such as those associated with butanoate and propanoate metabolism, characterizes the photic zone. Other types of metabolism, such as amino acid and glyoxylate metabolism, were also observed in the photic zone. Below the photic zone, at deeper layers, the water column showed chemolithotrophic pathways, such as methane metabolism and aromatic hydrocarbon degradation.

A general reconstruction of metabolic pathways that could be occurring within the marine samples analyzed in this study is depicted in Figure 10. Within the photic zone, there is a prevalence of fermentation processes, particularly butanoate and propanoate metabolism. However, it is important to point out the metabolism of amino acids and the degradation of aromatic hydrocarbons and alkanes due to their prevalence and abundance in accordance with the EZS of the enzymes described above.

The identification of enzymes with bioremediation potential in ocean samples as other molecules important for medical treatments and biotechnological uses is desirable because these enzymes are more tolerant to high pressure, salinity, and temperature than enzymes identified under other environmental conditions. We proved that our approach produced promising results in this regard. In this report, we describe some examples of the more significant cases to illustrate the scope of our method, leaving some enzymes and pathways less well explained. Moreover, metagenomic bins (Supplementary Table 9) encode a wide range of enzymes relative to oil and xenobiotic degradation pathways, particularly in the A04_AAIW and less in D18_MAX samples, as shown in Figures 7A,B, respectively.

## Conclusion

The amount of available biological data is continuously increasing; hence, the bioinformatics community has the golden opportunity to solve or retrieve hidden information from genomic and metagenomic data and gather information about microorganisms and gene functions that have been unexplored thus far. In this paper, we present a method for identifying the metabolic potential within metagenomic samples. Our proposed strategy explores the statistical properties of the enzymes collected from the samples, converting them into a baseline and allowing us to explore the overrepresentation of the enzymes catalyzing the reactions describing the metabolic potential of tested metagenomes. As a case study, we used two metagenomes collected in the GoM and sequences annotated by our group that were compared to reference metagenomes of the GEOTRACES project. This statistical analysis provided detailed examples of how our strategy generated clues about the metabolic network-defining pathways. Furthermore, the network reconstruction showed congruence between the identified pathways and the features of the sampled sites. Our results are promising, and we are aware that several steps in the sample treatment, sequencing, and bioinformatics pipelines employed for assembly and annotation may improve metabolic profile prediction. However, this strategy provided consistent results and a new approach for exploring the metabolic network of biomes.

## Data Availability Statement

The original contributions presented in the study are included in the article/Supplementary Material, further inquiries can be directed to the corresponding author.

## Author Contributions

AL and R-MG-R conceived and designed the methodology and analysis and wrote the manuscript. FG-G performed the network reconstruction and clustering analysis. AE-Z and LS performed the MG-RAST annotation. MS-O curated the essential enzymes to aid in the bioinformatics analysis, she assembled, binned, and annotated the genomes from the metagenomes, and prepared the Figures 2, 7, 10. FG-G and R-MG-R prepared the figures and tables. AE-Z, AS-F, EM, KJ, LS, and LP-L contributed to the improvement of the project and reviewed the final version of the manuscript. All authors contributed to the article and approved the submitted version.

## Conflict of Interest

The authors declare that the research was conducted in the absence of any commercial or financial relationships that could be construed as a potential conflict of interest.

## Publisher’s Note

All claims expressed in this article are solely those of the authors and do not necessarily represent those of their affiliated organizations, or those of the publisher, the editors and the reviewers. Any product that may be evaluated in this article, or claim that may be made by its manufacturer, is not guaranteed or endorsed by the publisher.

## Footnotes

1
https://github.com/OpenGene/fastp

2
https://cran.r-project.org/web/packages/MASS/MASS.pdf

3
https://github.com/baddstats/goftest

4
http://www.sbcs.qmul.ac.uk/iubmb/enzyme/

## Supplementary Material

Supplementary Figure 1 A04_AAIW metabolic network. The KGLM files were used to reconstruct the metabolic network at a depth of 1,000 m. The reconstruction was based on the significant identified enzymes (in red), connected by the compounds reported to represent a reaction. The network involved in the fluorouracil transformation is highlighted in a square.

Supplementary Figure 2 D18_MAX metabolic network. The KGLM files were used to reconstruct the metabolic network of the maximum fluorescence zone. The reconstruction was based on the significantly identified enzymes (in red), connected by the compounds reported to represent a reaction.

Supplementary File 1 Code example. The file presents the R functions employed to calculate the PDFs and EZS are shown.

Supplementary Table 1 Metadata. The table provides the SRA experiment identifiers for the reference and Gulf metagenomes, including the related MG-RAST identifier, geographical location, latitude, longitude, depth, library instrument, library layout, and PubMed references.

Supplementary Table 2 Probability density function describing the annotated enzymes in the reference. The table shows the PDF describing the behavior of each enzyme (EC) in the reference metagenomes (normal, log-normal, gamma, and Weibull distributions). The table also shows the parameters (Parameter 1 and Parameter 2), the rates on the references (m1–m19) and the observed rates on Rate D18_MAX and Rate A04_AAIW.

Supplementary Table 3 Probability density functions of aaRSs. The table presents the PDFs of the aaRSs of the reference. Enzymes were classified according to their classes (Woese et al., 2000) and KOs. Column 4 shows the number of genomes having the enzyme.

Supplementary Table 4 Probability density function of enzymes in metabolic pathways. The table shows the PDF describing the behavior of each enzyme in the reference metagenomes for which a metabolic pathway is described. The table presents from columns counts of enzymes with the same PDF within the pathways. Columns F–K show the enzymes’ frequency in the PDF found.

Supplementary Table 5 Equivalent *z*-scores of enzymes found in metabolic pathways. Enzymes with an assigned EZS were classified into the corresponding KEGG BRITE classes and metabolic maps.

Supplementary Table 6 Statistical properties of enzymes grouped into BRITE classes. A summary of the measures of the central tendency and quartiles is shown in the table with the enzymes grouped into the BRITE classes.

Supplementary Table 7 Enzymatic Commission equivalences. We show the enzyme names associated with ECs related to Figure 6 presented in the main text.

Supplementary Table 8 Enzymatic Commissions in clusters. We list the enzymes (EC: s) clustered within Figures 8, 9 from the main text.

Supplementary Table 9 Binned metagenome data. The features found for the metagenome bins in terms of completeness, contamination, and strain heterogeneity are listed. The final classification is set according to Checkm. The taxonomic classification of the binned metagenomes is also shown. Ensembles and annotations can be downloaded from Figshare https://figshare.com/articles/dataset/BINS_A04_AAIW_Gulf_of_Mexico/17132402 and https://figshare.com/articles/dataset/BINS_D18_MAX/17132417.

## Abbreviations

GoM: Gulf of Mexico
EC: Enzymatic Commission number
EZS: equivalent *z*-score
PDF: probability density function
Pi: inorganic phosphate.
