# Supplementary figures and images for: Definition of the Metagenomic Profile of Ocean Water Samples From the Gulf of Mexico Based on Comparison With Reference Samples From Sites Worldwide
Source: Front Microbiol. 2022 Jan 28;12:781497. doi: 10.3389/fmicb.2021.781497 (PMC8846951; doi:10.3389/fmicb.2021.781497)

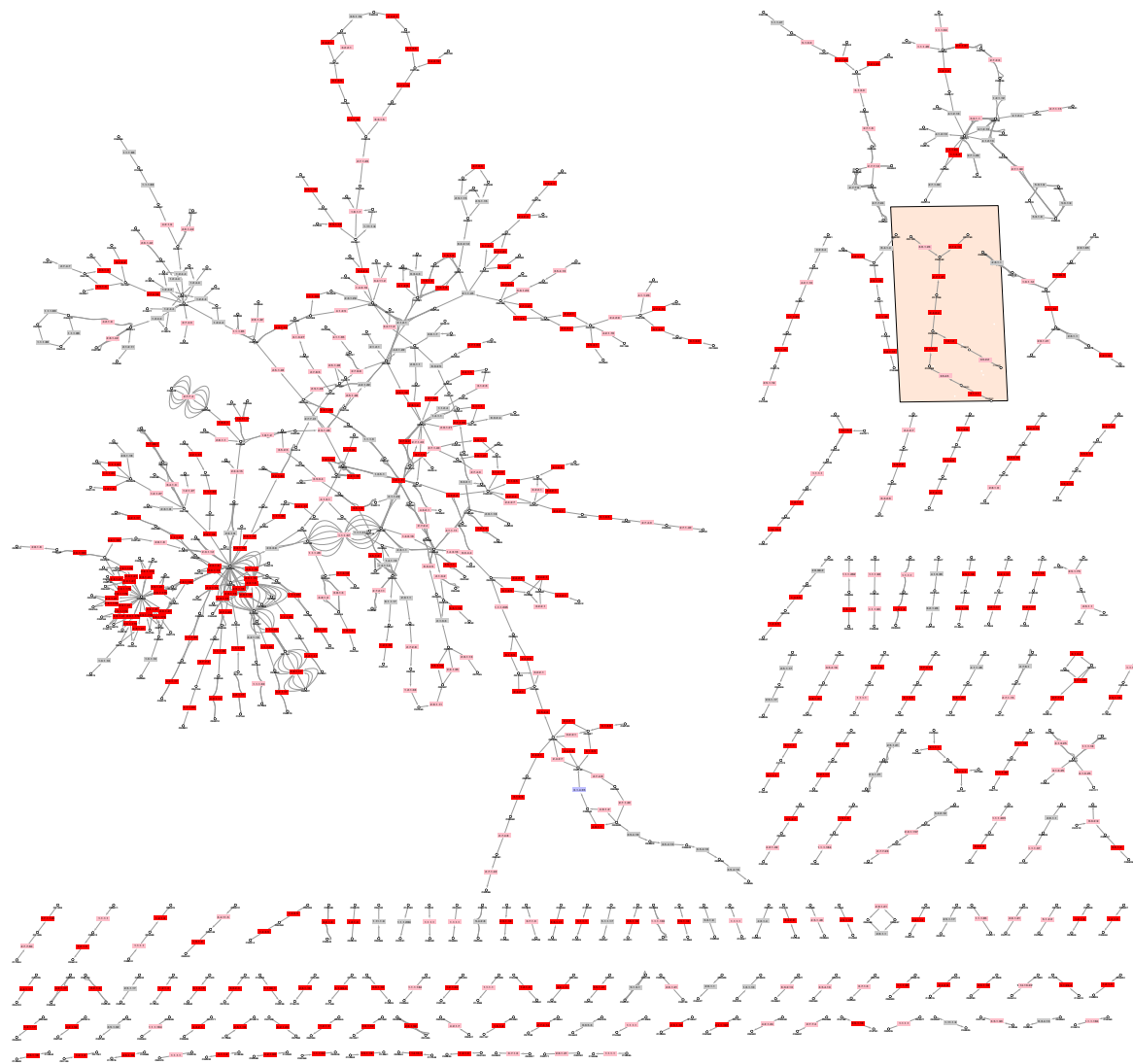

Supplement: Supplementary Figure 1 — A04_AAIW metabolic network. The KGLM files were used to reconstruct the metabolic network at a depth of 1,000 m. The reconstruction was based on the significant identified enzymes (in red), connected by the compounds reported to represent a reaction. The network involved in the fluorouracil transformation is highlighted in a square. [file Data_Sheet_1.PDF]

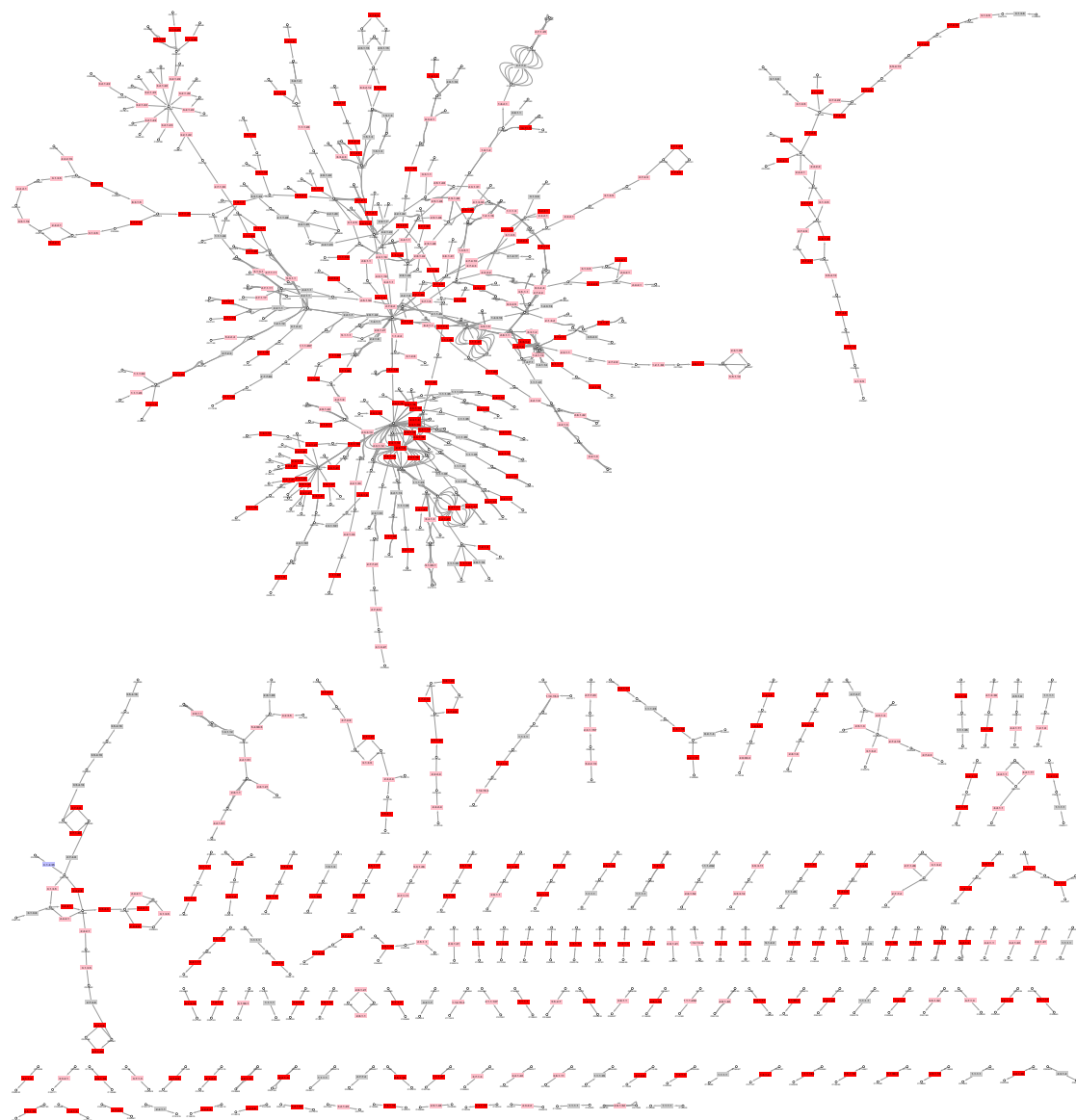

Supplement: Supplementary Figure 2 — D18_MAX metabolic network. The KGLM files were used to reconstruct the metabolic network of the maximum fluorescence zone. The reconstruction was based on the significantly identified enzymes (in red), connected by the compounds reported to represent a reaction. [file Data_Sheet_2.PDF]
