# Supplementary material for: Definition of the Metagenomic Profile of Ocean Water Samples From the Gulf of Mexico Based on Comparison With Reference Samples From Sites Worldwide
Source: Front Microbiol. 2022 Jan 28;12:781497. doi: 10.3389/fmicb.2021.781497 (PMC8846951; doi:10.3389/fmicb.2021.781497)
Supplement: Supplementary File 1 — Code example. The file presents the R functions employed to calculate the PDFs and EZS are shown. [file Data_Sheet_3.PDF]

We need to load the library MASS.

> library(MASS)

To fit the empirical data with a given probability density function, we execute
the command:

>fit <- fitdistr(y,densityFun)

where "y" is the vector of observed rates for the reference metagenomes (columns 9
to 27)
and "densityFun" is the name, enclosed in quotation marks, of the theoretical density
function to be used (column 2).

For instance, to fit the empirical data for enzyme EC:1.1.1.1, it is necessary to build
a numeric vector using the data from the reference.

> y <-
c(2256.11211141723,2036.76167389321,2108.67540510356,2074.5794364373,2261.2
8851256998,2047.97012821246,2188.18380743982,1890.96305150942,1834.996680
27666,2620.49781260845,2148.54298260032,2232.79244563527,2349.21317160634,
2031.08515604881,1805.03475766334,2159.6261053441,2058.58959877554,2148.77
56616374,2009.43805749653)

Now, by running

> fit <- fitdistr(y,"normal")

and then typing

> fit$estimate[1];fit$estimate[2] + return

you should obtain:

mean
2119.112
sd
182.5538

Which are the parameters of the fitted normal distribution. Note that these
parameters
differ slightly from those estimated using the mean and standard deviation of
the sample.

Other two examples:
Weibull (EC: 1.1.1.100)

> y <-
c(5214.5475844415,5335.5658573767,5600.28707015075,5060.31717345141,3535.37
27118165,4429.33074241299,3699.2547933467,5111.26797574209,5316.785253109
3,3836.47437841737,4942.82292718435,5017.51111378712,3151.94879347361,5324
.84901851652,5270.05683710636,3688.48519800077,4705.34765434409,4962.28048
478852,5048.10048590593)

shape
2.9281666
scale
80.5316369

gamma (EC: 1.1.1.107)
> y <-
c(84.0618695359785,112.459233527846,88.7014510751019,67.7721050015814,33.75
05748144773,47.6272122840106,34.4294655016756,118.507221211762,104.558215
40038,46.4530373455095,58.7033601803367,55.1926222516583,75.8490351370655,
95.5316126566926,107.442545099008,45.0547866205306,53.4698597084556,87.599
9670211889,42.0091579964432)

shape
6.72547089
rate
0.09401593

An gamma example, was use to illustrate how to calculate the equivalent Z score for the GoM metagenomes. In the case of metagenome D18 and EC:1.1.1.107, the observed rate
shown in the 5th column is 77.6599.   We enter the following command

> qnorm(pgamma(77.6599,6.72547089,0.09401593))

which gives the desired result

[1] 0.3440728

The call to the inner function "pgamma(.)" calculates the probability, with the specified parameters, of observing a rate equal to or lower than
observed
and corresponds to left hand side of eq (2) in the main text.

Next, using the quantile function for normal distribution qnorm(.) we obtain the z score.
Recall that when using qnorm, if mean or sd are not specified, they assume the default values of 0 and 1, respectively. The obtained z score if defined implicitly in eq (3).

Thus, the p-value associated with a given z score can be obtained with

the command:

>pgamma(77.6599,6.72547089,0.09401593)

For the A04 metagenome, the observed rate is 175.342 (column 7), typing.

> qnorm(pgamma(175.342 ,6.72547089,0.09401593))

we obtain the EZS

[1] 2.849127
